# The Association between Ethanol Elimination Rate and Hangover Severity

**DOI:** 10.3390/ijerph17124324

**Published:** 2020-06-17

**Authors:** Marlou Mackus, Aurora JAE van de Loo, Johan Garssen, Aletta D Kraneveld, Andrew Scholey, Joris C Verster

**Affiliations:** 1 Division of Pharmacology, Utrecht Institute for Pharmaceutical Sciences (UIPS), Utrecht University, 3584CG Utrecht, The Netherlands; marloumackus@gmail.com (M.M.); a.j.a.e.vandeloo@uu.nl (A.J.v.d.L.); j.garssen@uu.nl (J.G.); a.d.kraneveld@uu.nl (A.D.K.); 2 Institute for Risk Assessment Sciences (IRAS), Utrecht University, 3584CM Utrecht, The Netherlands; 3 Global Centre of Excellence Immunology, Nutricia Danone Research, 3584CT Utrecht, The Netherlands; 4 Centre for Human Psychopharmacology, Swinburne University, Melbourne VIC 3122, Australia; andrew@scholeylab.com

**Keywords:** alcohol, hangover, severity, ethanol, elimination rate

## Abstract

Assessments in blood and saliva suggests that the amount of ethanol present in the first hours after alcohol consumption and into the following morning is associated with hangover severity. The current analysis determines how ethanol elimination rate is related to hangover severity reported throughout the day. *n* = 8 subjects participated in two studies. The first was a naturalistic study comprising an evening of alcohol consumption. Hangover severity was assessed hourly from 10 a.m. to 4 p.m., using a 1-item hangover severity scale ranging from 0 (absent) to 10 (extreme). The second study comprised a highly controlled alcohol challenge to reach a breath alcohol concentration (BrAC) of 0.05%. Breathalyzer tests were conducted every 5 min until BrAC reached zero. The ethanol elimination rate, expressed in BrAC%/hour, was computed by dividing the peak BrAC (%) by the time to BrAC of zero (h). At 11:00, 13:00, and 14:00, there were significant negative partial correlations, controlling for estimated BrAC, between ethanol elimination rate and hangover severity. The findings suggest that drinkers with a faster ethanol elimination rate experience less severe hangovers. The observations should be confirmed in a larger sample of subjects who participate in a single study that assesses both hangover severity and ethanol elimination rate.

## 1. Introduction

The alcohol hangover refers to “the combination of negative mental and physical symptoms, which can be experienced after a single episode of alcohol consumption, starting when blood alcohol concentration (BAC) approaches zero” [1,2]. Although there is increasing research attention paid to the alcohol hangover, its pathology has not been fully elucidated [3,4,5]. As a consequence, no effective hangover treatment is currently marketed [6,7,8,9]. The relatively limited research attention to hangovers is of concern, because the hangover state is characterized by a variety of deleterious physical effects coupled with negative cognitive and mood changes [10]. These likely impact cognitive and psychomotor functioning [11], and daily activities including driving [12,13] and job performance [14]. To illustrate the societal impact of having hangovers, its annual economic costs due to absenteeism and presenteeism at work have been estimated $173 billion in US and $4 billion in UK [15,16].

Previous studies suggest that 25% of drinkers report not having hangovers, despite consuming large quantities of alcohol [17,18,19]. Directly comparing this subsample’s characteristics with individuals who are susceptible to having hangovers may shed light on the pathology of the alcohol hangover. The reason why some individuals are less susceptible to having hangovers is currently unknown. Previous studies have not found consistent significant differences in either urine biomarkers of alcohol metabolism [20,21], mental resilience [22] or in mood and demographic characteristics [23]. However, Van de Loo et al. found that, compared to hangover-resistant drinkers, individuals who report hangovers have a poorer (self-reported) immune fitness [24]. This observation was consistent across all BAC levels, independent of the amount of alcohol consumed. A subsequent survey, however, failed to demonstrate a significant correlation between hangover severity and perceived immune fitness [25].

In another study, Van de Loo et al. [26] compared ethanol concentrations of urine samples collected the morning following heavy alcohol consumption of hangover-susceptible and hangover-resistant drinkers. Of importance, the groups did not differ significantly on the amount of alcohol consumed (around 11 standardized drinks) and the estimated BAC (around 0.18%). Van de Loo et al. [26] found that urine ethanol concentrations were significantly lower in hangover-resistant individuals compared to hangover-susceptible drinkers. This observation suggests that the rate of the ethanol elimination is faster in hangover-resistant drinkers.

The current analysis directly investigated whether there is a relationship between ethanol elimination rate and hangover severity. Based on the observation that hangover resistant drinkers have lower urine ethanol concentrations during the hangover state, it was hypothesized that a faster ethanol elimination rate would be associated with experiencing less severe hangovers.

## 2. Methods

Data from *n* = 8 subjects that participated in both an acute alcohol challenge experiment [27] and a hangover study [23] were evaluated to determine if hangover severity was related to the ethanol elimination rate. Subjects were included if they were male or female healthy social drinkers between 18 and 30 years old, reported no physical or mental disease, and were non-alcoholic and/or nondependent drinkers. Participants were excluded from participation when a positive urine drug or pregnancy screen was obtained, and in cases where medicinal drugs were used (including over-the-counter pain killers), or consumed caffeinated drinks or foods on test days.

### 2.1. Study 1. Assessment of Ethanol Elimination Rate—Acute Study

The acute study [27] was a controlled experiment in which subjects completed an alcohol challenge to reach a peak BrAC of 0.05%. After arrival at the institute, participants consumed a standardized meal and body weight was determined. Using the Dräger Alcotest^®^ 7410*^Plus^* COM breathalyzer, it was confirmed that breath alcohol concentration (BrAC) was zero before the start of the experiment. The amount of alcohol (mL) needed to achieve a peak BrAC of 0.05% was calculated for each subject individually, applying a modified Friel formula [28]. The calculated amount of alcohol was mixed with orange juice to a 250 mL beverage, which was consumed by the subjects within a time frame of 5–10 min. Thereafter, every 5 min a breathalyzer test was conducted until subjects reached a BrAC of zero on two subsequent tests. The ethanol elimination rate, expressed in BrAC%/hour, was computed by dividing the peak BrAC (%) by the time until BrAC reached zero (h). The University of Groningen Psychology Ethics Committee approved the study (Approval number ppo-015-198), and all participants provided written informed consent before the start of the study.

### 2.2. Study 2. Assessment of Hangover Severity—Hangover Study

The other study [23] comprised a naturalistic study assessing hangover severity throughout the day after a typical night out consuming alcohol, compared with an alcohol-free control day. The study applied a naturalistic design to closely mimic a realistic and representative alcohol consumption session [29]. As they were not present, the researchers had no influence on the participants’ drinking, nor on any other aspect of their behavior. Demographic data collected from the subjects included age, sex, body weight, and height. Time period of drinking, types of alcoholic beverages consumed, and activities during drinking (e.g., dancing) were under voluntary control of the subjects and were recorded the day after. Test days started at 9 a.m. At time points 10.00, 11.00, 12.00, 13.00, 14.00, 15.00, and 16.00, subjects rated the overall severity of their hangover on an 11-point scale ranging from 0 (absent) to 10 (extreme) [30].

Previous night’s alcohol consumption was recorded (number of units and the total duration of drinking). An adjusted Widmark formula [31] was used to calculate estimated BAC, taking into account sex, body weight, the amount of alcohol consumed, and the duration of alcohol consumption. The University of Groningen Psychology Ethics Committee approved the study (Approval number ppo-015-002), and all participants provided written informed consent before the start of the study.

### 2.3. Statistical Analysis

The statistical analyses were performed using SPSS, version 25 (IBM Corp, New York, NY, USA). Mean and standard deviation (SD) were computed for each variable. The ethanol elimination rate was correlated with the overall hangover severity assessed at different timepoints throughout the day, by computing partial correlations (r_P_) and controlling for estimated BAC. The partial correlation controlled for possible confounding factors. The latter is important as the reported hangover severity must be interpreted in light of these various confounding factors. These include the amount of alcohol consumed, the duration of drinking, body weight, and sex, which are all included in the formula to compute estimated BAC [31].

Because of the relatively small sample size, a bootstrapping technique was used [32,33]. This statistical method aims to simulate the population distributions of the partial correlations (r_P_). To obtain an adequate resampling size [34], B = 10.000 bootstrapped samples (of *n* = 8 subjects each) were created by randomly drawing cases (resampling), with replacement, from the original sample. For each of the bootstrap samples the bootstrapped partial correlation, denoted as r_PB_, was calculated. The standard error (SE) represents the variability of the r_PB_s across the bootstrap samples, and the reported “bias” measure represents the deviation of the overall r_PB_ from the r_P_ that was obtained from the original sample [35]. The bias corrected and accelerated bootstrapped 95% confidence interval (BCa 95% CI_B_) was computed for each correlation [36]. The lower and upper limit of the BCa 95% CI_B_ can range from −1 to +1, with narrower BCa 95% CI_B_s implying greater precision. The r_PB_ is considered statistically significant in case the BCa 95% CI_B_ does not include zero (corresponding to a significance level of α = 5%).

## 3. Results

*n* = 8 participants (five men and three women) were used in the statistical analysis. Their demographic data is summarized in Table 1.

**Figure 1:**
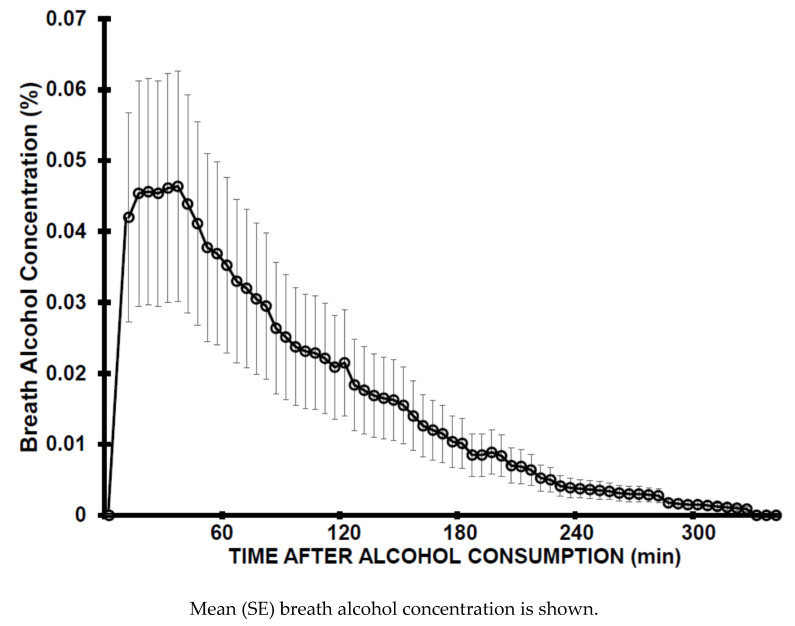
Breath alcohol concentration.

When participating in the acute study, subjects were one year older compared to participating in the hangover study. However, pairwise comparisons revealed that none of the other demographic data differed significantly between the two studies. Subjects consumed a mean (SD) of 33.7 (8.9) ml ethanol and reached a mean (SD) BrAC of 0.05 (0.02)% (See Figure 1). The mean (SD) ethanol elimination rate was 0.016 (0.002) BrAC%/h, with a range from 0.012 to 0.019 BrAC%/h.

**Figure 2:**
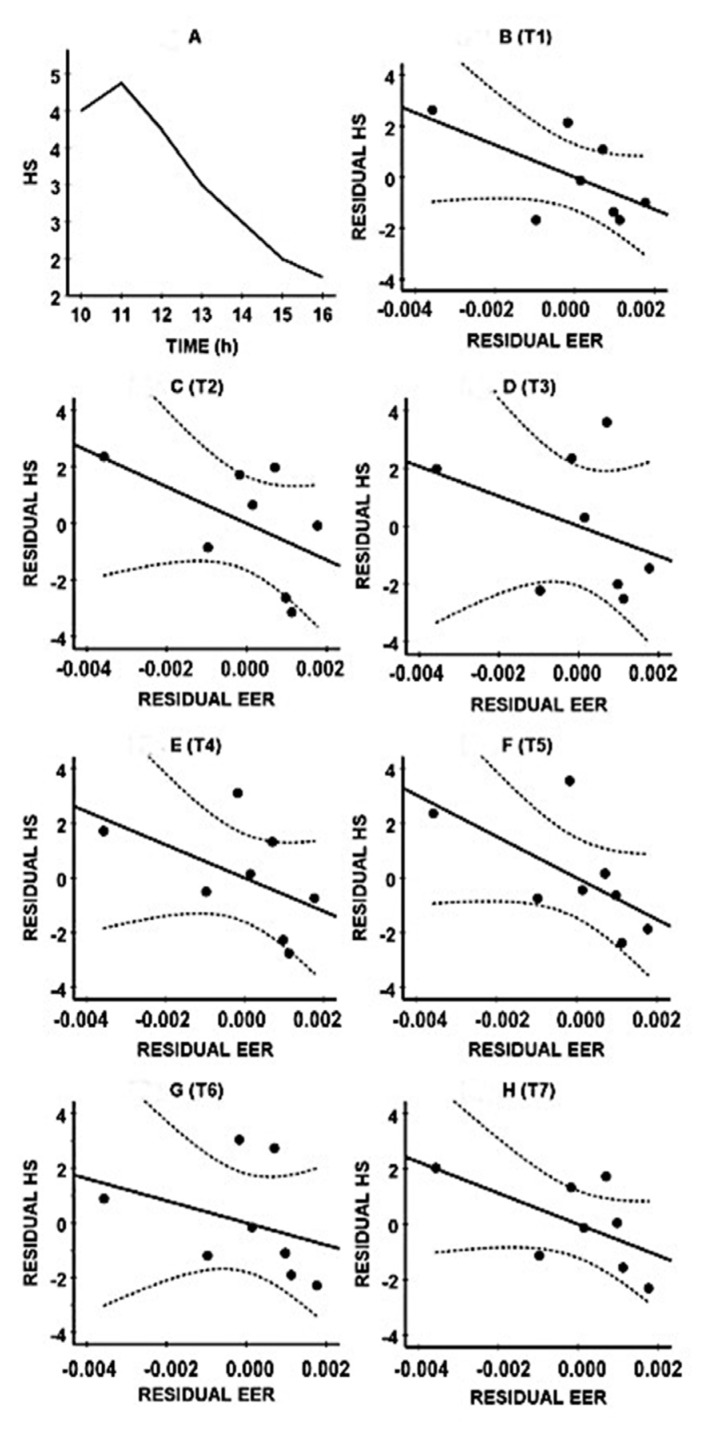
Relationship between ethanol elimination rate and hangover severity. (**A**): mean (SE) hangover severity; (**B**): T1 (10.00); (**C**): T2 (11.00); (**D**): T3 (12.00); (**E**): T4 (13.00); (**F**): T5 (14.00); (**G**): T6 (15.00); (**H**): T7 (16.00).

In the hangover study, on the alcohol test day, they reported consuming a mean (SD) of 11.8 (5.8) alcoholic drinks, resulting in an estimated mean (SD) BAC of 0.20 (0.10)%. Next-day hangover severity was greatest at 11.00 and then gradually decreased during the day (See Figure 2A). Figure 2B–H show the combined data of their ethanol elimination rate (BrAC reduction/hour) and reported hangover severity. The negative correlations suggest that those with lower hangover severity scores usually show a faster ethanol elimination rate. The bootstrapping analysis (See Table 2) revealed that the partial correlations were significant at T2 (10.00), T4 (13.00), and T5 (14.00).

Reported are the original partial correlations (r_P_), controlling for estimated blood alcohol concentration, and corresponding *p*-values). Bootstrapping was conducted with B = 10.000 samples. The bootstrapping correlations (r_PB_s) are significant (*p* < 0.05) if the 95% confidence interval (CI_B_) does not contain zero, which is indicated by *. Abbreviation: SE = standard error. Data from references [26,27].

Figure 2A shows the mean (SE) hangover severity over time. Figure 2B–H show the partial correlations, controlling for estimated BAC, between hangover severity and ethanol elimination at time point T1 (10.00) (Figure 2B), T2 (11.00) (Figure 2C), T3 (12.00) (Figure 2D), T4 (13.00) (Figure 2E), T5 (14.00) (Figure 2F), T6 (15.00) (Figure 2G), and T7 (16.00) (Figure 2H). The uninterrupted lines represent the partial correlations; the dashed lines mark the 95% confidence interval. Note: Ethanol elimination rate was computed as breath alcohol concentration reduction per hour. Abbreviations: HS = hangover severity, EER = ethanol elimination rate. Data from references [23,27].

## 4. Discussion

The current findings suggest that drinkers with a faster ethanol elimination rate experience less severe hangovers. There are several issues that should be taken into account when interpreting the data. First, the sample size of the current analysis was small. Although a bootstrapping technique was applied to account for this, the observations should be confirmed in a well-powered prospective study. Therefore, until a confirmation in a larger sample size comprising both sexes has been conducted, the current data and tentative conclusions should be regarded as preliminary. Second, the participants were drawn from a student population so were relatively young and healthy. It is therefore unclear to what extent the findings can be generalized to other populations. For example, increasing age is associated with higher ethanol elimination rates [37]. Additionally, relatively modest drinkers tend to have lower ethanol elimination rates compared to the current sample [37,38]. Third, the data on ethanol elimination and hangover severity was collected in two separate studies which might risk combining heterogenous data. Comparing the data, however, revealed that, besides an age difference of one year, demographics and drinking variables of the participants did not differ between the two studies. Thus we have some confidence that combining the data could be justified. However, future research should aim to replicate the current findings in a single study, assessing both ethanol elimination rate and hangover severity. Fourth, the ethanol elimination rate of 0.016 BrAC%/h, with a range from 0.012 to 0.019 BrAC%/h is in line with the fact that the sample consists of moderate to heavy drinkers, and in previous findings in samples with comparable alcohol intake levels. For example, Jones [38] reported an average ethanol elimination rate of 15 mg/100 mL/h, with a range from 10 to 35 mg/100 mL/h.

After consuming moderate amounts of alcohol (i.e., a BAC below 0.08%), ethanol elimination is primarily a zero-order process governed by alcohol dehydrogenase (ADH), in which ethanol is eliminated at a constant rate, independent of the amount of alcohol consumed [38]. Due to various factors (e.g., ethnicity, genetic variations in alcohol-metabolizing enzymes, and food consumption), this elimination rate shows some inter-individual variability between drinkers [37,38,39]. However, the ethanol elimination rate is found to increase when very high amounts of alcohol are consumed [40]. At higher drinking levels, ethanol is also eliminated via the microsomal ethanol oxidizing system (MEOS), governed by cytochrome CYP2E1. Via this so-called first order process, ethanol is eliminated at a dose dependent rate, which is faster when greater amounts of alcohol are consumed [38]. In the current analysis, it is assumed that the ethanol elimination rate assessed after the alcohol challenge to achieve a BrAC of 0.05% accurately reflects the ethanol elimination rate when larger amounts of alcohol are consumed. Although disputed elsewhere [41], here we embraced the assumption that the increase in ethanol elimination rate (due to simultaneous zero order and first order elimination) is proportional to the amount of alcohol consumed [42], therefore allowing a combination of the datasets from the two studies. Thus the ethanol elimination rate after BrAC 0.05% may be systematically lower (i.e., across all participants) compared to the ethanol elimination rate in the hangover study. It is however not likely that this systematic difference will significantly affect the outcomes of correlational analyses. However, given the inconsistent and limited amount of literature on this issue, it is advised that in future studies the ethanol elimination rate should be determined after alcohol consumption levels that correspond to the actual amount of alcohol consumed that results in a hangover, and preferably in a single study.

Still, there are some practical limitations to determine ethanol elimination in a hangover study, as participants will typically be asleep when on the descending limb of ethanol concentration. Therefore, in a single study, breathalyzer tests should preferably be replaced by (automated or manual) nocturnal blood sampling, which can be conducted during the night without awakening the participant or altering sleep patterns, to ensure a “normal” night of sleep [39,43]. A single assessment before going to bed and when waking up in the morning will likely not be effective, as in most drinkers BAC returns to zero during the night, i.e., when subjects are asleep. The latter implies that a combined study requires supervised sleep in a setting were blood sampling and sample storage is possible. As such, participants will not sleep in their usual environments (at home). Therefore, familiarization with the sleep unit is advised, as sleep in a new environment may affect both sleep quality and duration [44], which may impact the experienced hangover severity [45,46].

Finally, although the current sample comprised both men and women, it was too small to investigate potential sex differences. It is known that ethanol elimination rate is usually faster in women compared to men [37,38], and this is reflected in sex-specific elimination rates in the Friel formula [28] and the Watson formula [31]. A previous study could not identify profound sex-specific differences in the presence and severity of hangover symptoms when drinkers were classified according to their estimated BAC [47]. It is possible that sex-specific differences do become evident if drinkers are classified according to their ethanol elimination rate rather than the estimated BAC. Future studies should thus include a sufficient amount of participants to allow a comparison between men and women.

A strength of our findings is that all correlations between hangover severity and ethanol elimination rates are high and in the same direction. Moreover, they are consistently observed across all timepoints assessed during the day. The observations are also in line with previous research showing that the use of hangover treatments that claim to speed up alcohol metabolism is associated with reporting a reduced hangover severity [48,49,50]. Our findings have no direct implications for individual drinkers, because they will not know their ethanol elimination rate (unless they have been tested in the laboratory). However, the observed relationship between ethanol elimination rate and hangover severity is highly important for the general understanding of the pathology of the alcohol hangover. This could also have practical implications for the development of an effective hangover treatment, as the data suggest that treatments that are capable of speeding up alcohol metabolism may mitigate hangover symptoms.

## 5. Conclusions

Taken together, the current study showed a strong and consistent negative correlation between ethanol elimination rates and hangover severity assessed throughout the day. Future studies should confirm this observation in a larger sample size.

## Figures and Tables

**Table 1:** Demographics.

Demographics	Acute Study (2016)	Hangover Study (2015)	*p*-Value
Age (years)	21.4 (2.4)	20.4 (2.4)	0.005 *
Height (m)	1.78 (0.06)	1.78 (0.06)	0.685
Weight (kg)	67.6 (8.7)	67.1 (7.6)	0.499
Body Mass Index (BMI, kg/m^2^)	21.5 (2.6)	21.3 (2.5)	0.469
Usual Number of Drinks on Evening Out (units)	7.8 (2.9)	8.8 (3.2)	0.351
Number of Hangovers Per Month	2.4 (1.8)	2.1 (2.0)	0.345

Data are presented as mean (SD). Significant differences (*p* < 0.05) are indicated by *.

**Table 2:** Relationship between ethanol elimination rate and hangover severity.

Assessment	Original Sample				Bootstrapping Results			
Time	r_P_	*p*-Value	Bias	SE		r_PB_	Lower CI_B_ Limit	Upper CI_B_ Limit
10.00 (T1)	−0.607	0.148	0.069	0.403		−0.676	−0.952	+0.219
11.00 (T2)	−0.519	0.233	−0.009	0.308		−0.510 *	−0.857	−0.127
12.00 (T3)	−0.362	0.425	−0.020	0.386		−0.342	−0.921	+0.249
13.00 (T4)	−0.508	0.244	−0.036	0.272		−0.472 *	−0.922	−0.221
14.00 (T5)	−0.629	0.130	−0.021	0.272		−0.608 *	−0.973	−0.302
15.00 (T6)	−0.332	0.467	−0.066	0.366		−0.266	−0.972	+0.109
16.00 (T7)	−0.586	0.166	0.026	0.353		−0.612	−0.973	+0.050
